# A stepped wedge cluster randomized implementation trial to increase outpatient management of low-risk pulmonary embolism from the emergency department – the MEDIC ALERT PE study

**DOI:** 10.1186/s43058-025-00720-1

**Published:** 2025-04-02

**Authors:** Shawna N. Smith, Colin F. Greineder, Joshua Errickson, Jessica Burns, F Jacob Seagull, Keith E. Kocher, Jeffrey A. Kline, Jeffrey T. Kullgren, Michael S. M. Lanham, Sarah L. Krein, Geoffrey D. Barnes

**Affiliations:** 1 Department of Health Management and Policy, School of Public Health, University of Michigan, Ann Arbor, MI USA; 2 Michigan Program On Value Enhancement, University of Michigan Medical School, 2800 Plymouth Rd, B14 G214, Ann Arbor, MI 48109 USA; 3 Department of Emergency Medicine, University of Michigan Medical School, Ann Arbor, MI USA; 4 Center for Statistical Consultation and Research, University of Michigan, Ann Arbor, MI USA; 5 Center for Bioethics and Social Sciences in Medicine, University of Michigan Medical School, Ann Arbor, MI USA; 6 Department of Learning Health Sciences, University of Michigan Medical School, Ann Arbor, MI USA; 7 Department of Emergency Medicine, Wayne State University School of Medicine, Detroit, MI USA; 8 Department of Internal Medicine, Division of General Medicine, University of Michigan Medical School, Ann Arbor, MI USA; 9 Department of Obstetrics and Gynecology, University of Michigan Medical School, Ann Arbor, MI USA; 10 Center for Clinical Management Research, VA Ann Arbor Healthcare System, Ann Arbor, MI USA; 11 Frankel Cardiovascular Center, University of Michigan Medical School, Ann Arbor, MI USA

**Keywords:** Pulmonary embolism, Implementation science, Home-based treatment, Emergency department, Implementation frameworks

## Abstract

**Background:**

Home-based care for patients diagnosed in emergency departments (EDs) with low-risk pulmonary embolism (PE) is an evidence-based, guideline-recommended practice that is not widely adopted in the US. Few studies demonstrate how this care pathway can be implemented effectively or test whether implementation strategies can address known barriers. Further, prior studies have lacked diversity in population and health system type and did not integrate theory-informed implementation frameworks. Although essential for establishing the evidence base for safe home management of low-risk acute PE, these studies have thus fallen short of guiding broad dissemination and equitable implementation. To bridge this gap, we are conducting a pragmatic multi-site implementation trial, guided by implementation science theory and frameworks, across twelve diverse hospital settings to assess the effectiveness of new care pathways for patients with low-risk PE presenting to EDs.

**Methods/design:**

The study uses a cluster-randomized stepped wedge trial design to investigate a set of implementation strategies to support establishing low-risk PE pathways in 12 EDs. Clusters of three hospitals were randomly assigned to one of four start dates, staggered over a 12-month period. During an initial three-month pre-implementation period, we will work with site champions to identify key site personnel and understand site barriers and facilitators. We will then tailor the care pathway to local needs and capabilities. During the six-month active implementation period, we will provide coaching to help sites implement a multi-component intervention informed by behavioral economics intended to address multi-level (site, provider, patient) barriers and integrate the new care pathway for discharging low-risk PE patients. Sites are then followed for a minimum of 12 months post-implementation. Our primary aim is to assess the change in discharge rates of patients with acute PE pre- and post-implementation. Secondary and exploratory aims will assess change in patient safety outcomes along with other key implementation outcomes guided by the RE-AIM framework.

**Discussion:**

This study expands upon prior effectiveness research to tailor, implement, and robustly evaluate a multi-component implementation intervention for diverse health systems aiming to increase guideline-based outpatient management of low-risk PE. Broad-scale implementation in the US could avert up to 100,000 hospitalizations annually.

**Trial registration:**

Clinicaltrials.gov (NCT06312332), registered on March 13, 2024.

Contributions to the literature

- This stepped-wedge trial will use implementation science frameworks and a novel behavioral nudge to increase the use of home-based care for patients with low-risk acute pulmonary embolism
- The use of theory-informed implementation frameworks and behavioral economic interventions is unique in the design of a clinician-facing implementation intervention
- This study aims to improve evidence-based care across a diverse set of health systems following an Implementation Mapping process to adapt the implementation plan to local contextual needs

## Background

Pulmonary embolism (PE) is a frequent diagnosis in emergency department (ED) settings, impacting more than 250,000 Americans annually [1]. These patients utilize a high degree of health care resources, in particular, because up to 95% are admitted to hospitals [2]. However, research suggests that 25–40% of patients with acute PE in ED settings can be safely managed at home [3, 4]. In addition to reduced cost, outpatient management of low-risk acute PE reduces complications and aligns with patient preferences.

Leading society guidelines suggest that patients with acute PE at low risk of complications can be safely discharged for home-based care [5–7]. Guidelines advocate for providers' use of simple risk assessment tools to identify patients with acute PE at low-risk of complications. The most extensively studied risk tools include the Pulmonary Embolism Severity Index (PESI) [8], simplified PESI (sPESI) [9], and Hestia criteria [10], all of which quantify patient risk as a function of a patient’s demographics, comorbidities, and clinical presentation.

Guidelines also recommend the use of direct oral anticoagulants (DOAC) for patients with low-risk acute PE as DOAC medications are easy to administer and have robust evidence supporting their safety and efficacy [5–7]. However, even with these evidence-based guideline recommendations, outpatient management of acute PE in the United States (US) remains low. This starkly contrasts with care in Canada and parts of Europe, where patients with low-risk acute PE are routinely managed in the outpatient setting. As such, efforts are needed to determine how best to implement guideline-recommended outpatient management for patients with low-risk acute PE diagnosed in US EDs.

Several key barriers have been identified that may stymie US EDs from implementing care pathways that support home-based management of low-risk PE patients [8]. These barriers suggest that high-fidelity implementation of these care pathways may fail without thoughtful planning that addresses ED clinician lack of familiarity with outpatient trial results, ED clinician workflow, patient access to anticoagulation therapy, and rapid, reliable outpatient follow up. Further, behavioral economics may also inform promising strategies for ensuring that care pathways, once established at an institution, are used by ED providers.

A key barrier that has not been thoroughly considered is that ED clinicians commonly rely on fast-thinking (type I) heuristics to make rapid clinical assessment and treatment decisions. This is a necessary skill developed during training that allows ED clinicians to assess and treat critically ill patients rapidly. That same fast-thinking heuristic also maximizes their efficiency when caring for a large volume of patients with a wide range of clinical conditions. However, this fast-thinking heuristic can also impede the adoption of new evidence-based clinical practices (e.g., outpatient management of low-risk acute PE). Furthermore, a fast-thinking heuristic may constitute an important “blind spot” in the design of implementation strategies, as stakeholders are less likely to recognize or emphasize this heuristic’s contribution to decision-making as compared with other key barriers. As such, one crucial barrier to changing ED clinician behavior to improve discharge rates for low-risk PE patients is the need to alter ED clinicians’ well-established heuristic that associates an acute PE diagnosis with the need for hospital admission. Therefore, implementation strategies derived from behavioral economics (like pre-commitment, which asks clinicians to commit to following evidence-based practice, and point-of-care nudges, which provide just-in-time reminders when patients who can benefit from new practices or care pathways present) are promising implementation strategies to facilitate behavior change in the face of these ED clinician heuristics.

This study aims to test the implementation outcomes of a four-component intervention across twelve diverse EDs in the state of Michigan. We will evaluate the implementation of the care pathways to better understand the necessary components and test a strategy for scaling up pathway implementation, and also assess key safety outcomes to provide further evidence in support of outpatient management for patients with low-risk acute PE.

## Methods

### Study overview, design, and aims

We will use a pragmatic, stepped wedge cluster-randomized trial design to evaluate the implementation of a four-component implementation program to improve home-based treatment of low-risk acute PE for patients in 12 participating EDs across Michigan. Each of these EDs is an active participant in an ongoing quality improvement collaborative, the Michigan Emergency Department Improvement Collaborative (MEDIC). The MEDIC program received support from Blue Cross Blue Shield of Michigan to abstract data from the medical record that can be used by clinical champions at each site to engage in quality improvement.

To enhance the implementation of care pathways to improve home-based treatment for low-risk acute PE, we will utilize a process informed by implementation mapping to guide intervention tailoring at all 12 sites (Aim 1). Subsequently, we will employ a stepped wedge cluster randomized trial design to support sites in implementing their tailored multi-component intervention plan. Steps will include three sites at a time, with sites receiving six months of intensive implementation support and a further 12 months of follow-up support. Data collection will continue throughout the pre- and post-implementation periods. Consistent with a hybrid type III implementation-effectiveness study design, our primary and secondary outcomes will assess the adoption and other implementation outcomes informed by the RE-AIM framework [9] (Aim 2), and exploratory analyses will assess patient safety outcomes (Aim 3).

### Intervention: The care pathway for low-risk PE patients and associated implementation strategies

**Fig. 1:**
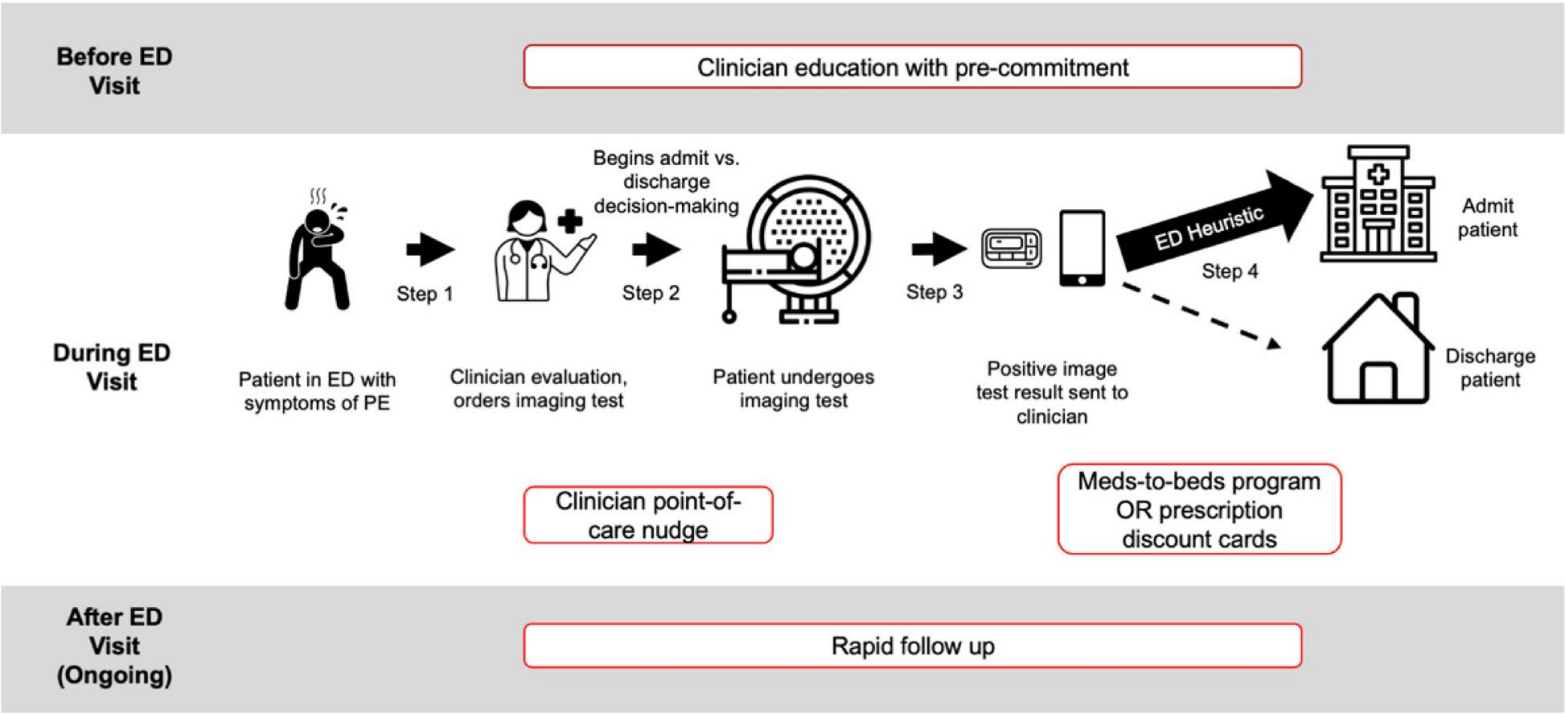
Current and home discharge care pathways for low-risk pulmonary embolism patients who present to the emergency department, with the four components of the care pathway implementation bundle indicated in red-lined boxes

This work aims to implement care pathways for low-risk PE patients at 12 sites across the state. This study is informed by pilot work that established both (1) the key components of the care pathway; and (2) a set of multi-level implementation strategies necessary to integrate that care pathway into the ED. Together, this care pathway “bundle” was designed to target specific barriers at the patient, provider, and site levels to the implementation of a safe and effective care pathway for low-risk PE patients. Figure 1 summarizes the care pathway for low-risk PE patients, including both the key pathway bundle components (described in more detail below) and the ED heuristics that these bundle strategies are targeting to disrupt.

### Pilot work

Prior to this multi-site project, we engaged key stakeholders at one academic medical center to understand the ED clinician workflow and identify key barriers to outpatient management of low risk acute PE. From these qualitative interviews, we identified that ED clinicians begin planning patient disposition (i.e., hospital admission versus home discharge) soon after their first clinical assessment of the patient. We also identified general familiarity with various acute PE risk tools, but a lack of detailed knowledge in how to calculate acute PE risk and the data supporting safe outpatient management. Furthermore, the ED clinicians identified that reliable access to anticoagulant medication and clinic follow up were key barriers to managing patients in the outpatient setting. We then assembled a multi-disciplinary stakeholder group to select and tailor implementation strategies that directly addressed each of these barriers. Over a 6-month period, these strategies were implemented within a single ED with broad stakeholder support and notable increase in the use of outpatient management for patients with low-risk acute PE.

### Care pathway “bundle” components

The care pathway bundle includes four key components, each designed to address one specific barrier to outpatient management of acute PE (Table 1). The first two strategies address the barriers that ED clinicians identified themselves. The second two strategies address key barriers identified by the study team but not explicitely stated by individual ED clincians and stakeholers.

**Table 1:** Specification of Implementation Strategies (Site- and Study-Provided)

	**Care pathway “bundle” strategies** ***(up to sites to offer)***					**Centralized study-offered implementation support** ***(offered to all sites)***						
	**Deliver structured educational program**	**Clinician pre-commitment**	**Point-of-care nudge**	**Facilitate Medication Access**	**Rapid follow-up**	**Needs assessment and pathway tailoring**	**Prepare structured educational program**	**Coaching for CQI champion**	**Build a coalition to support implementation**	**External peer support**	**Data audit**	**Provide technical assistance with FAQ and EHR components**
**Primary Actor(s)**	Clinical champions; Study staff	Clinical champions	Clinical champions; Site IT staff	Clinical champions	Clinical champions	Study staff	Study staff	Study staff	Study staff	Study staff; Clinical champions	Study staff, MEDIC staff	Study staff; Clinical champion
**Action(s)**	Organize an educational session with clinicians and deliver the educational content prepared by the study team	Design & offer opportunity for clinicians at site to pre-commit to using low-risk PE discharge pathway	Establish point-of-care nudge to help providers identify low-risk PE patients (e.g., EHR-integrated interruptive alert or checklist)	Establish a pathway for providing rapid and reliable access to anticoagulant medications upon ED discharge	Establish a pathway for rapid follow-up with an outpatient clinician shortly after ED discharge	Assess site needs & determinants; help sites tailor the low-risk PE pathway to site needs & capabilities	Prepare educational materials that address knowledge gaps, provide baseline data, inform about site-specific barriers, and detail the workflow package components	Offer phone-based, individualized coaching to site clinical champions to address barriers to care bundle adoption	Engage site champions & stakeholders to identify internal stakeholders to join a working group coalition that will lead the low-risk PE discharge intervention at each site	Offer opportunities for implementing sites to reflect on the implementation effort, share lessons learned, & support learning external to their individual site	Monitor & disseminate data on use of low-risk PE discharge pathway	Provide clinical champions with access to FAQ documents and EHR tools for point-of-care nudge implementation
**Targets of the action**	Frontline ED clinicians	Frontline ED clinicians	Frontline ED clinicians	Frontline ED clinician	Frontline ED clinician	Clinical champions	Frontline ED clinicians	Clinical champions	Clinical champions	Clinical champions & stakeholders	Clinical champions & stakeholders	Clinical champion
**Temporality**	Offered at least once during implementation phase (typically last month)	Following educational session	System set up during implementation phase; messages delivered at time of clinical encounter	Occurs upon decision to discharge from ED but prior to patient leaving the ED	Occurs upon decision to discharge from ED but prior to patient leaving the ED	Pre-implementation phase	Offered at least once during implementation phase (typically last month)	Implementation phase	Throughout implementation phase	Throughout the six-month implementation period	Post implementation phase	Throughout the six-month implementation phase
**Dose**	One session	Once following each educational offering	Once per low-risk PE clinical encounter	Once per low-risk PE clinical encounter	Once per low-risk PE clinical encounter	Once	Once prior to site roll-out; upon request (up to two more times) during post-implementation	Every two weeks during implementation phase; monthly for the first six months of the post-implementation phase, quarterly thereafter	Every two weeks during implementation phase; monthly for the first six months of the post-implementation phase, quarterly thereafter	At least once during the implementation phase; every three months post-implementation phase	Monthly for the first six months of the post-implementation phase, quarterly thereafter	Anytime during the implementation phase
**Implementation outcome(s) affected**	Acceptability, adoption, implementation fidelity, reach, maintenance	Adoption, implementation fidelity	Reach, adoption, implementation fidelity, maintenance	Adoption, implementation fidelity, maintenance	Adoption, implementation fidelity, maintenance	Feasibility, appropriateness, adoption, implementation fidelity, maintenance	Acceptability, adoption, implementation fidelity, reach, maintenance	Adoption, implementation fidelity, maintenance	Adoption, implementation fidelity, maintenance	Adoption, implementation fidelity, maintenance	Adoption, maintenance	Adoption, implementation fidelity, maintenance
**Justification**	Clinician training builds knowledge & capability among ED clinicians to ensure know how & when to discharge low-risk PE patients	Commitment mechanisms help clinicians adopt new practices by encouraging them to commit prior to a time-sensitive clinical encounter when heuristics may prevail	Reminders help clinicians adopt new practices reminding them about available pathways & commitments to use them	Addresses an important barrier identified by ED clinicians	Addresses an important barrier identified by ED clinicians	Helps the study team understand barriers to be addressed by implementation efforts & tailors care pathway components to what is feasible for site	Clinician training builds knowledge & capability among ED clinicians to ensure know how & when to discharge low-risk PE patients	Provides hands-on, tailored support to address barriers & help further tailor effort	Ensures that clinical champions feel empowered to support effort & minimizes turnover &/or replacement time	Peer learning & collaboration improves implementation by allowing sites to learn from one another	Sharing data helps sites see how/whether they are improving & how their rates of utilization compare to other sites	Provide sits with technical resources so that they do not need to re-create EHR tools or resources for rapid follow up clinicians

Providers often expressed concerns about patients not being able to receive timely access to medications and/or necessary follow-up care. As such, two key elements of the intervention are establishing programs for (1) facilitating immediate medication access; and (2) rapid patient follow-up post-discharge.

- *Facilitating Immediate Medication Access:* To address concerns about medication availability, sites implementing the care pathway will need to implement either a meds-to-beds program, wherein a pharmacist delivers anticoagulant medications to the patient in the ED for ambulatory use (often a 30-day supply) prior to discharge, or provide prescription drug cards to ensure patients are able to access the necessary medications, irrespective of financial status, access to transport, etc.
- *Rapid Follow-up Program:* To ensure safe outcomes, low-risk PE patients discharged to home should be seen for follow-up by an outpatient provider within seven to ten days of discharge. As part of the care pathway, sites must ensure that rapid follow-up options are available and integrated into the discharge process. The exact form of this follow-up care can be tailored to site capabilities, with options for providing this follow-up, including dedicated follow-up clinics, clinician appointments, or telehealth follow-up from nurses/pharmacists.

In addition to these pathway components that address ED clinician-specified barriers, our pilot work also revealed several implementation strategies that are likely necessary to ensure high-fidelity, widespread adoption and reach of the pathway that ED clinicians did not explicitly specify. These include:

- *Structured Education Program:* Site-wide education, ideally led by regional/national leaders in PE care, is necessary to ensure that all providers engaging with the pathway are aware of (1) what home management of low-risk PE entails; (2) how best to estimate acute PE risk and appropriateness for home-based management (i.e., use of PE risk scores); and (3) the safety data underlying the use of these pathways for discharge to home.
- *Clinician Pre-commitment and Point-of-care Nudge:* To disrupt the ED clinician’s fast thinking heuristic, we recommend sites develop two complementary strategies: first, following completion of the educational program, clinicians should “pre-commit” to discharging patients, for example, by publically agreeing to consider risk in determining whether hospital admission is necessary, and discharge appropriate low-risk patients to home. Second, timeliness of implementation strategies is key for maximizing the broad adoption of an innovation such that delivery of a point-of-care nudge should be provided to clinicians when they are making care decisions for patients with PE, not after they have already made a decision. Specifically, this means the delivery of a point-of-care nudge to consider home-based care management, if medically appropriate, before the clinician is alerted to a positive computed tomography (CT) finding since their fast-thinking heuristic will immediately link that positive CT result with the need for hospital admission. An alert at the time a clinician enters an admission order, or even when they enter the heparin anticoagulant order, may be too late as the clinician has already “anchored” on an admission disposition decision and is less likely to change behavior.
  o The preferred method for this point-of-care nudge is to embed an acute PE risk score calculator into the electronic health record. In our pilot work, this was accomplished by building a calculator of the PESI into the Epic™ electronic health record, as has been accomplished in other settings [10].

### Centralized implementation support offered by the study team

**Fig. 2:**
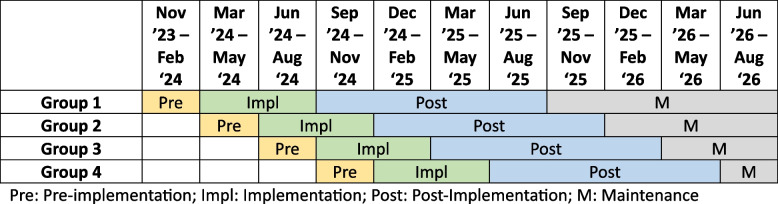
Stepped Wedge Design. Pre: Pre-implementation; Impl: Implementation; Post: Post-Implementation; M: Maintenance

To support our sites in implementing this new care pathway, we will also offer centralized implementation support, focused on scaling up training materials (high quality versions of which can be challenging/resource intensive to develop), helping site troubleshoot challenges that arise as they work through site-level implementation, and learning from other sites that are also actively implementing the care pathway. Strategy selection was informed by use of a modified implementation mapping approach [11], wherein data on barriers and facilitators was collected from our pilot site, change objectives related to these barriers were then identified, and a set of potential, appropriate implementation strategies were selected, each linked to one or more change objectives. From this set, we then selected an initial set of implementation strategies that addressed the most crucial barriers and were feasible. These strategies are described briefly below and full, initial specifications [12] are included in Fig. 2. Many of these strategies will utilize the sites’ existing clinical champions who currently collaborate with other clinical champions from across the state to implement evidence-based care strategies, such as reducing unnecessary imaging and reducing hospitalization for low-risk patients presenting with chest pain. Four strategies specifically are key to our team’s work:

- *Needs Assessment and Workflow Package Tailoring:* Study staff will conduct interviews with key site members to ascertain needs and then work with site members to tailor the care pathway to site capacity and capability.
- *Structured Provider Eduation Program:* We will offer all participating sites and their care provider teams educational sessions covering home management of low-risk PE. Content and delivery modality will be tailored to site needs and will be made available through various modalities, including in-person/virtual sessions and asynchronous materials. The Educational Program will cover the key elements of the pathway and the safety data underlying home management of PE. It will also include site-specific details about how to engage with the care pathway.
- *Building a Coalition to Support Implementation:* The study team will help the CQI champion identify and convene key stakeholders for implementing the care pathway at their site, including stakeholders related to establishing rapid follow-up and medication access, offering training opportunities for care teams, and implementing electronic health record (EHR) configuration.
- *Coaching for CQI Champion:* To help the CQI champion address barriers identified during the needs assessment and/or new barriers that emerge during the implementation process, study staff (including a clinical ED expert) will be available to meet with sites on a biweekly basis to talk about progress and help them strategize. This model of coaching is loosely based on models of External Facilitation that have proven successful for implementing other new care pathways [13–15].
- *Facilitating External Peer Support:* As an extension to site-specific activities, study staff will also work to facilitate communication between sites within a particular step to encourage peer support and information sharing, especially as it relates to addressing common or similar barriers.
- *Technical Assistance, including Support for EHR Builds*: We will offer sites the option to transfer the pilot site’s PESI calculator to their electronic health record system (if they also use Epic®) or will provide the underlying logic to facilitate the local build of a similar calculator and alert in other EHR systems.
- *Data Audit and Feedback:* Data on rates of discharge of low-risk PE patients will be provided to sites on a quarterly basis for purposes of monitoring progress through the existing MEDIC data registry.

### Study design

The centerpiece of our study is a 12-site cluster-randomized stepped wedge trial evaluating the impact of the implementation strategies on the implementation of the care pathway, as well as the effectiveness of care pathway “bundle” implementation on effectiveness outcomes. Each site was considered a “participant.” The cluster-randomized stepped wedge trial design involves random and sequential crossover of clusters comprising three sites apiece from usual care to implementation until all sites receive the intervention. This design allows each site to serve as its own control. We can use this design to assess intervention effects and distinguish them from temporal trends. Continuous recruitment with short exposure minimizes patient exposure to both intervention and control conditions.

### Site eligibility and recruitment

The study will be conducted across 12 centers participating in the MEDIC program [16, 17]. MEDIC comprises over 50 hospital emergency departments, collectively processing more than 2 million visits annually, involving over 1200 emergency physicians. The MEDIC registry records data from over 2500 patients with acute pulmonary embolism (PE) each year. These sites encompass a mix of urban and non-urban, as well as academic and community settings, with more than half serving patient populations with over 20% non-white demographics, facilitating the study of implementation disparities.

A subset of MEDIC sites were identified as eligible for participation in this trial. Specifically, sites were considered eligible to participate if:

1. They had an active MEDIC clinical champion who could facilitate implementation activities;
2. They had an acute PE volume of ≥ 80 diagnoses in the 2022 calendar year.

All eligible sites were contacted by one of the study leaders (CFG), an ED clinician, to confirm eligibility and interest in study participation. Most sites were contacted through their existing MEDIC clinical champion by phone or email between January and May 2023. Of the 15 total sites that were contacted, 12 agreed to participate.

### Implementation mapping

Randomized sites in each step will proceed through several implementation phases. During the first phase, pre-implementation (three months), the study team will work with each site’s CQI Champion to designate a project leader (anticipated to most often be the current CQI Champion) and relevant stakeholders (e.g., pharmacists, social workers, administrators, information technology experts) who will also comprise the stakeholder coalition supporting implementation. The project leader and key stakeholders will then be tasked with tailoring both the care pathway components and study-provided implementation strategies to the local site needs. To inform this tailoring, the study team will conduct a needs assessment via semi-structured qualitative interviews with the identified stakeholders. Data from these interviews will be used to develop site-specific implementation mapping, specifically by updating the list of previously established determinants, identifying and/or tailoring specific care pathway components (e.g., identify entity responsible for rapid follow-up, tailor nudge modality), and assessing the feasibility of planned implementation strategies and, if needed, adapting specification (e.g., dosage or timing of coaching, modality of educational modules).

All identified stakeholders or other site decision-makers will be contacted by email and/or phone to complete an interview with the study team. To update implementation materials in a timely fashion, rapid qualitative inquiry will be used to identify new barriers and update the site-specific mapping of strategies to barriers. Reports on identified barriers/facilitators and planned implementation activities will be shared with each site. The FRAME and FRAME-IS frameworks [18, 19] will also be used to document modifications, including initial planned modifications to centrally provided implementation support.

### Evaluation of implementation and effectiveness outcomes

The full trial will roll out implementation support to 12 sites in four site groups, with each site advancing through four study phases (Fig. 2). As described above, the initial *pre-implementation phase* will focus on assessing site needs, identifying stakeholders, and tailoring the care pathway bundle to site needs. During the second phase, *implementation*, we will support sites in implementing the care pathway bundle, including implementing the site-provided implementation strategies specified in Fig. 2. The goal of this six-month phase is for sites to be ready to “go live” with the care pathway prior to phase end. This phase also comprises the most intensive study team-provided implementation support, including delivery of educational modules to care teams towards the end of this phase. The third study phase is *post-implementation*, which will last 12 months. During this phase, sites will continue to receive some support from the study team, but the expectation is that the care pathway “bundle” has been implemented and providers are beginning to utilize it. Sites will also begin to receive data audits from the study team during this time, showing their rates of PE discharge and change over time. Finally, the fourth phase will assess the *maintenance* of care pathway implementation and use. Given staggered study entry, time in this phase will range from three months (Group 4) to 12 months (Group 1).

A Data Safety and Monitoring Board will be comprised of three members who are free from competing interests and the sponsor, who report to the institutional review board with any findings of concern.

#### Randomization

Given interdependencies between certain sites (e.g., part of the same health system, use of a single EHR), it was not feasible to randomly assign individual sites to steps, as is best practice in stepped wedge designs. To maximize independence between clusters but accommodate these interdependencies, we created four groups of three sites each (e.g., three sites belonging to the same health system) and then randomized the order in which groups would begin rollout. This pragmatic design strategy also meant that we were not able to stratify rollout based on key baseline data (e.g., ED volume of low-risk PE patients).

## Measures and outcomes

### Outcomes

The RE-AIM implementation evaluation framework guided outcome selection [9].

### Adoption (primary outcome)

Our primary outcome will measure the change in the proportion of all acute PE patients discharged from the ED without hospitalization post-implementation vs. pre-implementation.

### Reach (secondary outcome)

For reach, we will examine the change in the proportion of patients with acute PE who qualify as low-risk (defined as Pulmonary Embolism Severity Index class I or II [20]) that were discharged post-implementation vs. pre-implementation. We will also examine demographic and comorbidity characteristics of the discharged patients (vs. not) to assess whether reach of the care pathway was equitable or whether it introduced or exacerbated disparities in care.

### Implementation Fidelity (secondary outcome)

Fidelity will be defined as implementation of the care bundle pathway components, including the site-provided implementation strategies, as planned. As noted above, FRAME [18] and FRAME-IS [19] will be used to document planned and ad hoc modifications to both the care pathway and implementation strategies (either site or study team-delivered). We will also quantitatively assess the level of implementation fidelity to the five care pathway “bundle” components described above: (1) rapid follow up; (2) medication access; (3) delivery of education; (4) clinician pre-commitment; and (5) EHR nudge. Each component will be rated by study staff using a scale of 0–3 (0 – not implemented; 1 – minimally implemented or implemented with low fidelity; 2 – mostly [but not completely] implemented or with minimal alternations affecting fidelity; 3 – fully implemented with high fidelity).

*Maintenance (secondary outcome*).

Maintenance will be defined as trends in discharge of PE patients following the completion of the 12-month post-implementation period.

### Effectiveness/Patient Safety (exploratory outcomes)

As well-implemented care pathways that discharge appropriate PE patients should be just as safe, if not safer, than care pathways that admit PE patients, effectiveness of the care pathway will be assessed through an examination of three patient safety outcomes, all assessed at 30 days post-discharge: (1) return to the ED, (2) any bleeding that leads to health care system interaction, and (3) all-cause death.

### Other measures

#### Organizational readiness for change

To assess baseline organizational difference in site readiness for change, we will also assess organizational readiness for change using the validated 20-item Organizational Readiness for Change Assessment (ORCA) during our pre-implementation meetings with the site clinical champions [21]. All three domains (evidence assessment, context assessment, and facilitation assessment) will be included this assessment.

### Data collection

In addition to the semi-structured interview data collected during the pre-implementation phase for Aim 1 analyses (described above), two other sources of data will be central to this evaluation. Clinical data and outcomes, including for our primary outcome of care pathway adoption, will be attained via the existing central MEDIC registry. Study staff reports will inform evaluations of implementation fidelity and organizational readiness for change.

### MEDIC registry data

Data collection for the majority of our implementation and clinical effectiveness outcomes will be obtained for analysis through the existing central MEDIC Data Registry. All MEDIC sites, including the 12 study sites, currently contribute electronically extracted data into this registry. This includes data from all ED visits with imaging ordered to evaluate for possible acute PE (primarily computed tomography [CT]). For our purposes, results of the CT scan along with ICD-10 diagnosis codes (I26) will be used to identify a cohort of patients with acute PE. Additional data collected for each case include key demographics, comorbidities, medications and other treatments administered, and disposition data (i.e., hospital admission, home discharge). These data undergo regular audits by the MEDIC team (distinct from our study team) to ensure data accuracy and completeness. Data on key outcomes will be available from the pre-implementation phase through the end of the maintenance phase (a minimum of three months after the 12-month post-implementation phase).

### Study team data

#### Organizational readiness for change

At baseline, study team members involved with the needs assessment will collectively complete an ORCA evaluation [21] on behalf of each site to assess differences in perceived organizational readiness for change. One study team member will provide an initial assessment and at least one additional team member will evaluate ratings for concordance and cross-site consistency in ratings. Disagreements in ratings will be brought to the full study team for final rating decisions.

### Implementation fidelity

Study team members will be trained in using the FRAME [18] and FRAME-IS [19] tools to report planned and ad-hoc modifications to care pathway components, as well as study-provided implementation support. Modifications will be tracked on both tools separately by site as appropriate throughout the pre-implementation, implementation, and post-implementation phases, with modifications queried with sites at least every three months.

Quantitative assessments of implementation fidelity will be assessed by study staff at three time points: (1) end of implementation period; (2) six months into the post-implementation period; and (3) at the end of the post-implementation period. While assessments at the end of the implementation period will be considered primary for purposes of analyses, later assessments allow for reporting of sites that may achieve full or high-fidelity implementation of key care pathway components after the end of the implementation phase; they also allow for a assessment of sites’ abilities to maintain care pathway components. Study team assessments will follow the same process as for the ORCA above, involving a minimum of two central study team members that are actively engaged in communication with sites as to implementation progress.

### Analyses

Analyses will follow an intent-to-treat approach, encompassing all eligible patients presenting before and after study participation at all sites. We anticipate an increase in the mean proportion of acute PE patients discharged without hospitalization from 12 to 25% across all sites.

### Primary outcome

To assess changes in the proportion of patients with acute PE discharged from the ED rather than admitted to the hospital, we will employ mixed-effects logistic regression to analyze the data. The dependent variable will be a patient-level measure of hospital discharge (binary yes/no). The primary independent variables will be a measure of pre-post implementation state, a time variable, and a categorical variable of the group assignment. We will include an interaction between the categorical group assignment variable and the pre-post implementation state variable. Fixed effects covariates will include key patient characteristics (e.g., age, sex, race/ethnicity, insurance status, comorbidity score) and random effects for the site. Individual sites will be included as a random effects variable in the model. In the primary analysis, we will exclude the 6-month implementation period in order to best compare pre- vs. post-implementations states. In an exploratory analysis, we will include a 3-phase analysis of the pre-implementation, active implementation, and post-implementation phases.

In a secondary analysis, we will also include data from non-participating control sites. These sites will be assigned to the pre-intervention state during the entire time period. Any site that does not care for adult patients (e.g., pediatric hospital) or patients with acute PE (e.g., psychiatric hospital) will be excluded from this analysis.

### Fidelity-informed Adoption (exploratory measure)

To account for variations in fidelity to intervention components across different EDs, we will conduct an exploratory analysis focusing on the degree of implementation fidelity at an individual site level and the association with adoption. This analysis will replicate the primary adoption analysis but will also incorporate a categorical sum of the five intervention components (range 0–12) from the implementation fidelity assessment. If an association is found between the degree of intervention fidelity and site-level adoption, then we will explore the four individual components of the intervention and their association with the adoption outcome measure. These analyses will shed light on the significance of individual intervention components in driving outcomes.

#### Patient safety outcomes

These will be assessed among all patients with acute PE managed without hospitalization. Specifically, we will report the percent of patients who experience (1) return to the ED, (2) any bleeding that leads to healthcare system interaction, or (3) all-cause death within 30 days of the ED visit. A Data Safety and Monitoring Board will review safety data throughout the trial.

#### Sample size and power

Based on preliminary MEDIC registry data, we anticipate approximately 580 acute PE patients per site over the trial period, providing ample power for all outcomes. With a complete cluster-randomized stepped-wedge design, we'll have over 99% power to detect our hypothesized increase in the proportion of acute PE patients managed without hospitalization from 12 to 25%. Sensitivity analyses will ensure robustness across different scenarios.

## Discussion

This pragmatic, stepped-wedge implementation trial aims to implement and evaluate a four-component intervention to increase the use of evidence-based outpatient management for patients presenting to the ED with a low-risk acute PE. If shown to be effective, this trial will provide a model for implementing similar strategies at hospital ED’s across the US as well as provide a framework for other implementation efforts in the ED setting.

This study represents a critical advancement in the field of implementation science for acute PE management by incorporating several innovative elements. First, unlike previous efforts focusing primarily on effectiveness outcome measures, this study deeply integrates theory-informed implementation and de-implementation frameworks. To guide the implementation process, this study pioneers the use of Implementation Mapping [11] in the ED setting, offering a structured approach to intervention development and tailoring. By applying this approach across diverse sites, this study aims to establish a model for multi-site collaborative-based quality improvement and implementation efforts broadly across a wide range of clinical diagnoses and management domains.

Second, this study directly addresses the heuristics commonly used by ED clinicians in disposition decision-making. Through innovative strategies derived from behavioral economics, such as pre-commitment and point-of-care nudges, this study aims to facilitate behavior change and improve the appropriateness of clinical decision-making.

Two unique features of this study deserve further discussion. First, is the conduct of this implementation trial within an existing quality improvement collaborative. These networks often represent diverse health systems and have existing data collection infrastructure that reduces the burden of data management for the research team. In this case, the MEDIC collaborative has an ongoing effort to optimize CT use for patients suspected of having PE. As such, there is a culture of healthcare quality improvement around the diagnosis of acute PE that facilitates interest and participation in this project. However, no ongoing project within each of these centers addresses hospitalization versus outpatient acute PE management, minimizing potential contamination. Given that all MEDIC sites are already collecting and contributing data to the MEDIC data registry, no additional patient-level data is required for participation in this stepped-wedge implementation trial. Conducting implementation trials within an independently funded collaborative reduces costs for all involved parties: the primary research team reduces infrastructure costs while the collaborative benefits from rigorous implementation science research provided by external funding (in this case, the National Heart, Lung, and Blood Institute) that lowers the barriers to practice change. This is a promising model for low-cost/high-value implementation trials in the future.

The second unique feature of this study is the innovative approach to measuring implementation fidelity. While the FRAME and FRAME-IS tools [18, 19] are well described in the implementation literature, few studies have adopted a categorical assessment of implementation. The quantitative assessment of implementation fidelity provided by these tools can be integrated into a multivariable regression model to help identify which adaptations may have a greater or lesser influence on key outcome measures.

Our study has important limitations. First, while our study includes 12 diverse hospital EDs across the state of Michigan, these sites may not represent the entire diversity of all hospital EDs in the US. Furthermore, practice variation from state-to-state and between the US and other countries may limit the generalizability of these findings. Second, data collection will rely on the existing MEDIC collaborative data registry. While this data is audited for accuracy and completeness, it does not have robust post-ED data included. Our safety analysis will focus on 30-day events using a data matching protocol with Medicare, Medicaid, and private insurer claims data when available. However, not all patients will have complete post-ED claims for these analyses.

In conclusion, innovative implementation strategies are needed to change ED clinician behavior and increase the use of evidence-based, guideline-recommended outpatient management of low-risk acute PE. This stepped-wedge trial will evaluate a four component intervention across 12 diverse hospital EDs participating in the MEDIC quality collaborative. If found to be effective and broadly adopted, this multi-component implementation program will provide a framework for nation-wide adoption of this high-value, evidence-based practice in the United States.

## Data Availability

The quantitative datasets are managed by the MEDIC quality collaborative and are not available for public access due to data use agreements within the network. Qualitative datasets will be made available upon reasonable request.

## Abbreviations

CQI: Collaborative Quality initiative
CT: Computed Tomography
DOAC: Direct Oral Anticoagulant
ED: Emergency Department
HER: Electronic Health Record
FRAME: Framework for Reporting Adaptations and Modifications – Expanded
FRAME-IS: Framework for Reporting Adaptations and Modifications to Evidence-based Implementation Strategies
ICD-10: International Classification of Diseases, Tenth Revision
MEDIC: Michigan Emergency Department Improvement Collaborative
ORCA: Organizational Readiness to Change Assessment
PE: Pulmonary Embolism
PESI: Pulmonary Embolism Severity Index
RE-AIM: Reach, Effectiveness, Adoption, Implementation, Maintenance (framework for evaluation)
sPESI: Simplified Pulmonary Embolism Severity Index
